# Cultivation of *Chlorella vulgaris* and *Arthrospira platensis* with Recovered Phosphorus from Wastewater by Means of Zeolite Sorption

**DOI:** 10.3390/ijms16024250

**Published:** 2015-02-16

**Authors:** Giorgos Markou, Orily Depraetere, Dries Vandamme, Koenraad Muylaert

**Affiliations:** 1 Department of Natural Resources Management and Agricultural Engineering, Agricultural University of Athens, Iera Odos 75, Athens 11855, Greece; 2 Laboratory Aquatic Biology, KU Leuven Kulak, E. Sabbelaan 53, Kortrijk 8500, Belgium; E-Mails: Orily.Depraetere@kuleuven-kulak.be (O.D.); Dries.Vandamme@kuleuven-kulak.be (D.V.); Koenraad.Muylaert@kuleuven-kulak.be (K.M.)

**Keywords:** biomass, cyanobacteria, microalgae, nutrient recycling, phosphorus, wastewater treatment, zeolite

## Abstract

In this study, zeolite was employed for the separation and recovery of P from synthetic wastewater and its use as phosphorus (P) source for the cultivation of the green microalga *Chlorella vulgaris* and the cyanobacterium *Arthrospira* (Spirulina) *platensis*. At P-loaded zeolite concentration of 0.15–1 g/L, in which P was limited, the two species displayed quite different behavior regarding their growth and biomass composition. *C. vulgaris* preferred to increase the intracellular P and did not synthesize biomass, while *A. platensis* synthesized biomass keeping the intracellular P as low as possible. In addition under P limitation, *C. vulgaris* did display some little alteration of the biomass composition, while *A. platensis* did it significantly, accumulating carbohydrates around 70% from about 15%–20% (control). Both species could desorb P from zeolite biologically. *A. platensis* could recover over 65% and *C. vulgaris* 25% of the P bounded onto zeolite. When P-loaded zeolite concentration increased to 5 g/L, P was adequate to support growth for both species. Especially in the case of *C. vulgaris*, growth was stimulated from the presence of P-loaded zeolite and produced more biomass compared to the control.

## 1. Introduction

Microalgae and cyanobacteria are a potential source of biomass for the production of various chemicals for the food, pharmaceutical, industrial and bio-energy sector [1,2]. For the production of their biomass, a series of nutrients, such as carbon, nitrogen, phosphorus, potassium *etc.*, are needed in adequate amounts. Among the nutrients phosphorus (P) is one of the most significant and essential for the cultivation of microalgae and cyanobacteria [3]. Considering that P fertilizers are produced using fossil phosphate rocks, which are not renewable and are a resource that will be depleted in the near future, the recycling of nutrient from wastewaters is a significant way for sustainable biomass production.

Several wastewaters types are identified as potential nutrient sources for microalgae and cyanobacteria cultivation, including municipal, industrial and agro-industrial wastewaters [4]. However, these waste streams beside the desired nutrients may contain some other contaminants which can not only negatively affect the microalgae and cyanobacterial growth but may also reduce biomass quality, rendering the direct use of the wastewaters as cultivation medium or as supplement in the medium problematic [5,6]. Therefore in some cases it may be necessary to separate nutrients from the wastewater and transfer them to a cultivation medium.

In a previous work [7] zeolite was employed for the separation and recovery of ammonium from wastewaters and its supplementation as nitrogen source for the cultivation of the cyanobacterium *Arthrospira platensis*. It was shown that the separation and recovery of nutrients through the process of sorption of nutrients onto a surface and their recovery and application as nutrient in the cultivation medium is a promising approach for the production of biomass.

In natural environments and wastewaters, P is presented in various forms, such as orthophosphate, polyphosphate, pyrophosphate, metaphosphate and their organic complexes. Microalgae and cyanobacteria take up P mainly in the form of orthophosphate [8]. Orthophosphate is an anion, which is physically adsorbed on the zeolite. However, the affinity of natural zeolite for anion uptake is very low (<0.2 mg-P/g zeolite) because the permanent negative electrical charge of the zeolite’s framework [9]. In a previous work (submitted for publication) the chemical pre-treatment of zeolite with Ca(OH)_2_ resulted to an about 40-fold increase of the P adsorption capacity. After the chemical pre-treatment the zeolite was added in an artificial wastewater to adsorb P in the form of orthophosphate. The P mass which was finally bounded to the zeolite was 10.3 mg-P/g zeolite. Aim of the present study was to investigate the capability of using chemically pre-treated zeolite as a sorbent for P recovery from artificial wastewater and its application as P source for the production of two economically important autotrophic microorganisms, namely the green microalga *Chlorella vulgaris* and the cyanobacterium *Arthrospira platensis*.

## 2. Results

### 2.1. Desorption of Phosphorus

**Figure 1:**
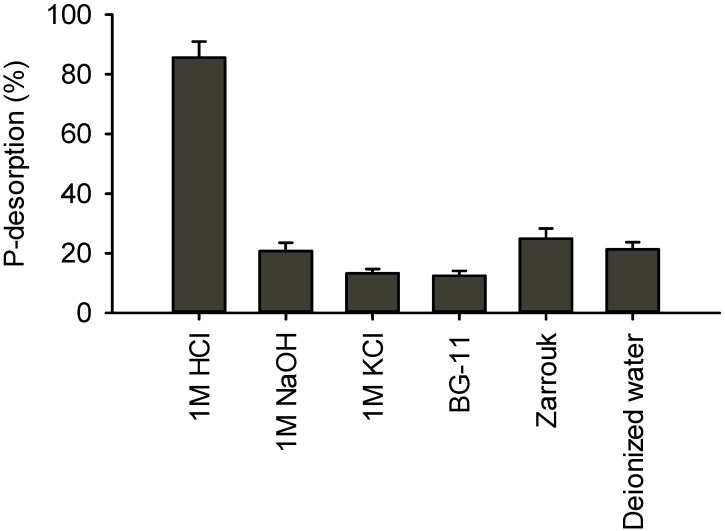
Desorption of P from P-loaded zeolite using various desorption solutions. P-loaded zeolite concentration 2.5 g/L, desorption duration 24 h.

Desorption of P on various solutions, including the two cultivation media, was investigated in order to determine the percentage of the bounded onto zeolite P that can be recovered through physical/chemical desorption. Desorption of bounded P was performed using various types of solutions, such as HCl, NaOH, KCl, deionized (DI) water and the two cultivation media BG-11 and Zarrouk, which were used for the cultivation of *C. vulgaris* and *A. platensis*, respectively. As shown in Figure 1, the most effective solution for P desorption was the acid (HCl), which was capable to desorb P at an efficiency higher than 85%. This indicates that the bounded P requisite acidic conditions to be desorbed. DI was capable to desorb around 20% of sorbed P, a fact that means that this portion of P was sorbed mainly physically and therefore was bounded through reversible bindings. Solutions of NaOH and KCl were not so effective like HCl, and they were capable to desorb 21% and 13%, respectively. Between the cultivation media, Zarrouk displayed a higher capability to desorb P (≈25%) than BG-11 (≈12%), probably due to the presence in higher concentrations of various ions (such as potassium, sodium *etc*.), which contributed to a higher ion-exchange process. The ion-exchange of potassium or sodium with calcium affected desorption of P which was bounded to calcium (as Ca–P complex).

**Figure 2:**
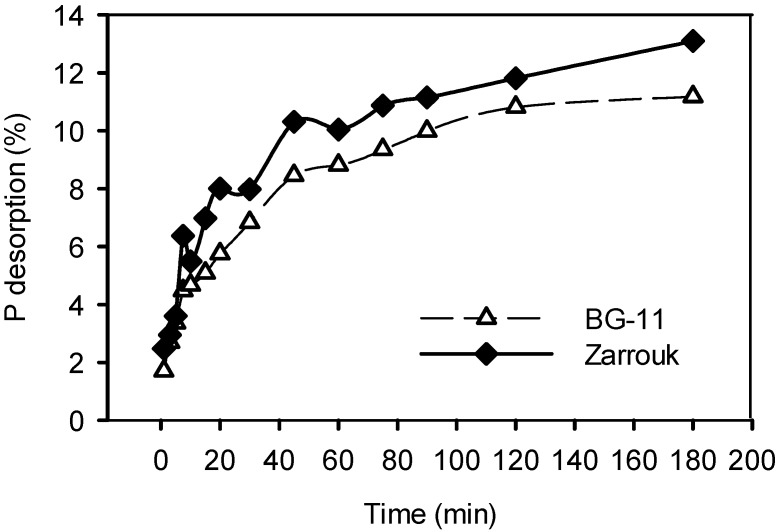
Desorption kinetics of P from P-loaded zeolite into BG-11 and Zarrouk cultivation media. P-loaded zeolite concentration: 2.5 g/L.

The kinetics of P desorption (Figure 2) into BG-11 cultivation medium, revealed that P desorption is a relative fast process and that most of the P was desorbed in the first 180 min. Desorption in the cultivation medium even after 72 h, reached about 12%. However, in the Zarrouk medium, desorption was slower and only after 72 h (data not shown) it was capable to reach its maximum desorption percentage of 25.5%. In other words, desorption equilibrium into Zarrouk medium needed more time to be established but with higher final percentage that in BG-11 medium.

### 2.2. Cultivation of C. vulgaris and A. platensis with P-Loaded Zeolite

#### 2.2.1. Cultivation with 0.15–1.00 g/L P-Loaded Zeolite

**Figure 3:**
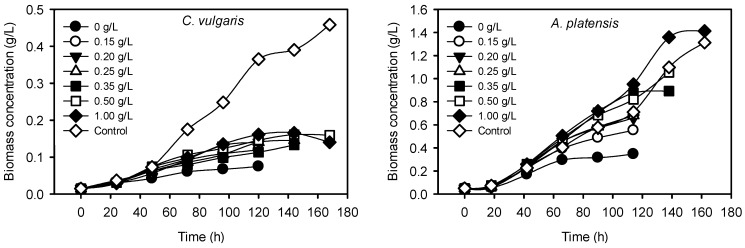
Biomass production during the cultivation of *C. vulgaris* and *A. platensis* in cultivation media supplemented with 0.15–1.00 g/L P-loaded zeolite as P source.

**Figure 4:**
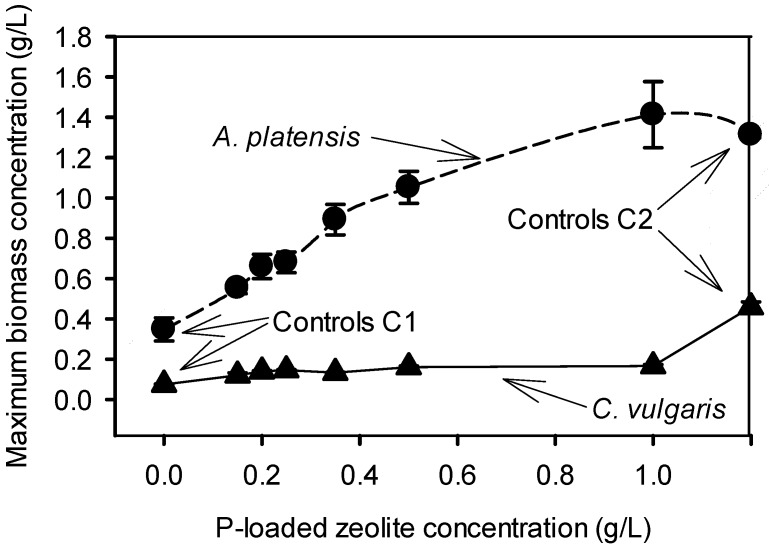
Relation between P-loaded zeolite concentration and maximum biomass density. Controls C1 refers to the culture grown without P-loaded zeolite and without P and controls C2 refers to those that were grown with replete P and without P-loaded zeolite.

To investigate the capability of *C. vulgaris* and *A. platensis* to grow utilizing the bounded P in the zeolite, a series of cultures was performed using P-loaded zeolite (with a P load of 10.3 mg-P/g zeolite) in concentration ranged from 0.15 to 1.00 g/L. As shown in Figure 3, in both species the supplementation of the cultivation media with P-loaded zeolite had a positive effect on the biomass production in comparison to the cultures without P (neither in the form of P-loaded zeolite nor in the form of K_2_HPO_4_). However, *A. platensis* displayed an impressively higher growth efficiency producing biomass as same as the control cultures (with 0.5 g/L K_2_HPO_4_), while in contrast *C. vulgaris* was not able to grow well and the biomass production was about four times lower than the control cultures (with 0.4 g/L K_2_HPO_4_). Clearly, the biomass production of *A. platensis* increased gradually and proportionally with the increase of P-loaded zeolite, while *C. vulgaris* had almost the same maximum biomass concentration for the whole range of P-loaded zeolite applied (Figure 4).

**Figure 5:**
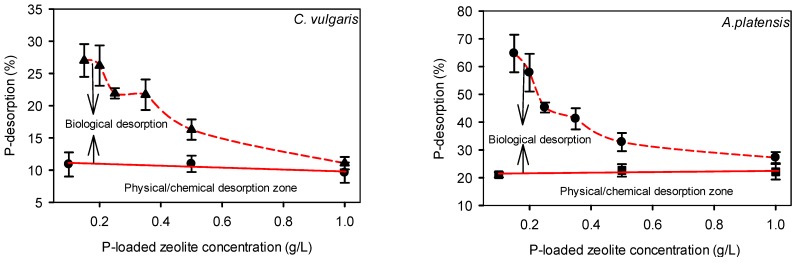
P-recovery of bounded P onto zeolite. Physical/chemical desorption zone refers to the mass of P desorbed from zeolite into control medium without microalgae.

The concentrations (0.15–1 g/L) of P-loaded zeolite were chosen so that the P source will be gradually limited to see in what extend the microorganisms were able to utilize the zeolite-bounded P. For all the cultures after the third day no P were detected in the cultivation medium, showing that the physically/chemically desorbed P were all removed. Note that, especially in the BG-11 medium, in which the chelating agent (Na_2_EDTA) is in low concentration, and due to the fact that the pH of the culture medium increased and reached 9.5 (due to release of OH^−^ during photosynthesis), the formation of calcium or magnesium phosphate complexes and their precipitation is highly possible. However, for the intracellular P estimation the biomass was washed with DI water for at least 2 times, a fact that should dissolve and remove any P precipitation. Therefore the intracellular P reflects the real P uptake but may not reflect the real desorbed P from the zeolite. The intracellular P of both species was higher than the portion of physically/chemically desorbed P into the cultivation medium (Figure 5), suggesting that the taken up P was desorbed from zeolite biologically (energetically).

The degree of the biological desorption for each species is shown in Figure 5. In both species the P recovery increased as the concentration of P-loaded zeolite decreased, indicating that at stronger P limitation the microorganisms had a stronger P desorption and uptake capability. P-recovery for *C. vulgaris* was in general significantly lower than that of *A. platensis*. However the intracellular P in *C. vulgaris* was higher than that of *A. platensis*. The biomass P content is species- and condition-dependent and varies greatly between species, but also for each species it varies among the different cultivation conditions [10,11]. However in all cases, when P is critically limited, the P content in biomass tends to decrease. For *Chlorella* and *Arthrospira* species, P contents of 2.3 mg-P/g up to 19 mg-P/g [10,12], and 1.8 mg-P/g up to 10 mg-P/g [10,13,14], respectively, were reported.

**Figure 6:**
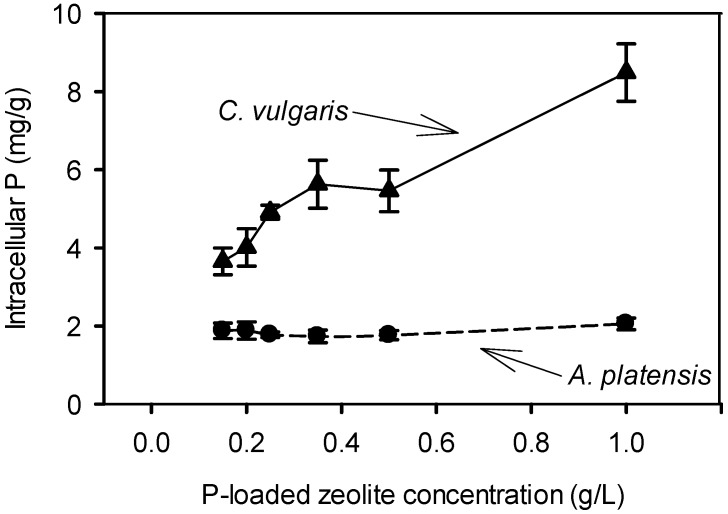
Intracellular P against concentration of P-loaded zeolite.

The observations of the present study indicate a different metabolic strategy between the two species studied of producing biomass in relation to the available P. As can be seen in Figure 6 *C. vulgaris* increased the intracellular P repressing growth, while in contrast *A. platensis* gave priority in biomass synthesis keeping the intracellular P as low as possible. In addition, *C. vulgaris* seems to prefer to keep almost unaltered the cell biochemical composition against biomass production, while *A. platensis* prefers to synthesize biomass by changing significantly its biochemical composition, accumulating carbohydrates up to 70% from about 15%–20% (Table 1).

#### 2.2.2. Cultivation with 5 g/L P-Loaded Zeolite

**Figure 7:**
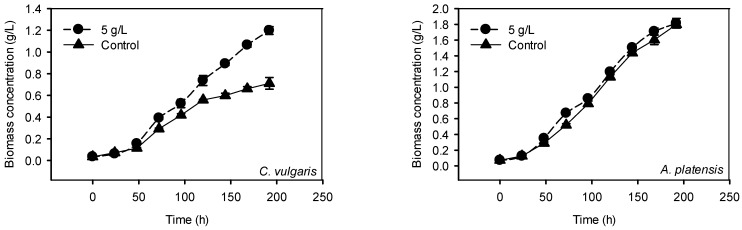
Biomass production during the cultivation of *C. vulgaris* and *A. platensis* in cultivation media supplemented with 5 g/L P-loaded zeolite as P source.

In a second series of cultures, P-loaded zeolite was supplemented to the media in concentration of 5 g/L (51.5 mg bounded P per liter) in order to investigate the behavior of *C. vulgaris* and *A. platensis* with replete P source. As shown in Figure 7, the growth of *C. vulgaris* was significantly enhanced by the supplementation of 5 g/L of P-loaded zeolite in comparison with the control cultures (0.4 g/L K_2_HPO_4_). However, the biochemical composition of *C. vulgaris* differed between the cultures (Table 2). Intracellular P, carbohydrate and protein contents were lower, while lipid content was higher in cultures with 5 g/L than that in control. These results show that the other compounds sorbed to zeolite such as ammonium or micro-nutrients enhanced the growth of *Chlorella*.

In contrast, *A. platensis* cultivated with 5 g/L of P-loaded zeolite displayed the same growth as that of the control, and the biochemical composition was almost the same in both cultures (Figure 7 and Table 2). In both cases, it is demonstrated that P-loaded zeolite in concentration of 5 g/L is an adequate source of P for biomass production.

**Table 1:** Biochemical composition of *C. vulgaris* and *A. platensis* cultivated with 0.15–1.00 g/L P-loaded zeolite as P source.

P-Loaded Zeolite	Intracellular P (mg-P/g)		Carbohydrates (% Dry Biomass)		Proteins (% Dry Biomass)		Lipids (% Dry Biomass)	
	*C. vulgaris*	*A. platensis*	*C. vulgaris*	*A. platensis*	*C. vulgaris*	*A. platensis*	*C. vulgaris*	*A. platensis*
0.15	3.7 ± 0.3	1.9 ± 0.2	31 ± 2	70 ± 3	16 ± 2	19 ± 2	11 ± 2	7.2 ± 0.5
0.2	4.0 ± 0.5	1.9 ± 0.2	28 ± 1	71 ± 3	16 ± 1	21 ± 2	13 ± 2	5 ± 2
0.25	4.9 ± 0.2	1.78 ± 0.07	36 ± 2	71 ± 2	15.8 ± 0.4	18 ± 1	12 ± 4	5.9 ± 0.8
0.35	5.6 ± 0.6	1.7 ± 0.2	37 ± 1	63 ± 4	16 ± 2	16 ± 1	16 ± 2	6.0 ± 0.5
0.5	5.5 ± 0.5	1.8 ± 0.1	42 ± 3	61 ± 8	16 ± 2	18.5 ± 0.6	19 ± 2	5.0 ± 0.8
1	8.5 ± 0.7	2.1 ± 0.2	48 ± 5	52 ± 12	21 ± 2	29 ± 2	24 ± 5	12.5 ± 0.6
Control (with K_2_HPO_4_)	7.8 ± 0.6	6.2 ± 0.4	43 ± 1	14 ± 2	25 ± 1	32 ± 1	21 ± 2	8.8 ± 0.8

**Table 2:** Biochemical composition of *C. vulgaris* and *A. platensis* cultivated with 5 g/L P-loaded zeolite as P source.

Species	Culture	Intracellular P (mg-P/g)	Carbohydrates (% Dry Biomass)	Proteins (% Dry Biomass)	Lipids (% Dry Biomass)	Chlorophyll α	Chlorophyll β	Carotenoids
*C. vulgaris*	5 g/L	9.4 ± 0.3	35.9 ± 0.8	22 ± 1	25 ± 2	2.1 ± 0.3	0.5 ± 0.1	0.60 ± 0.05
	Control	13 ± 2	40 ± 1	35 ± 4	18 ± 1	2.17 ± 0.06	0.56 ± 0.03	0.62 ± 0.02
*A. platensis*	5 g/L	6.3 ± 0.5	20 ± 1	53 ± 2	12.4 ± 0.6	1.11 ± 0.07	–	0.32 ± 0.01
	Control	6.8 ± 0.5	18 ± 1	53 ± 3	10.8 ± 0.5	1.07 ± 0.06	–	0.32 ± 0.02

## 3. Discussion

The use of wastewater streams for microalgal and cyanobacterial biomass production is gaining interest worldwide. This is mainly attributed due to the potential of microalgae and cyanobacteria to produce biomass that can be used as feedstock for biofuels production [15,16]. In biofuel application of biomass, the contamination of the biomass with unwanted particulates from wastewater should not be an issue and a wide range of qualities could be used for the production of biofuels. However, microalgal and cyanobacterial biomass display some interesting biochemical characteristics (content of proteins, essential fatty acids, anti-oxidant, vitamins *etc.*), which render them an attractive source of high value products that could be used in the nutrition of humans and animals [17]. But, although it is not documented so far in the literature, the use of wastewater as cultivation media or as supplement may contaminate the produced biomass with various unwanted wastewater particulates (such as bacteria, viruses, dissolved or suspended solids, heavy metals, persistent organic pollutants or other toxic compounds). This fact will lower the value of the biomass and will exclude it from using it in the nutrition sector. It is however significant to find solutions on the production of biomass for food and feed in sustainable way. The reuse of nutrients from wastewaters is of particular significance, however, we should find ways to separate these nutrients from other contaminants present in the wastewater before being used as cultivation medium or as supplement.

In the present study zeolite was used for the sorption of phosphate from artificial wastewater to cultivate two economically important species, namely the green microalga *C. vulgaris* and the cyanobacterium *A. platensis*. The chemically pre-treated zeolite is able to adsorb substantial amounts of P, about 10–15 mg-P/g zeolite (submitted for publication). The sorbed P, however, is not easily desorbed from the zeolite. In pure water as well as in microalgae cultivation medium, only about 15% to 25% of the adsorbed P is desorbed (Figure 1). Desorption can be increased under acid conditions, but the addition of acid would render the medium unsuitable for microalgae cultivation. Microalgae, however, appear to enhance desorption of P from zeolite. In the presence of microalgae, desorption of P from the zeolite increased significantly compared to a control medium without microalgae (Figure 5). This suggests that P is desorbed through a biological mechanism. This was particularly the case when P-loaded zeolite was supplied in low concentrations, which suggests that biological P desorption is triggered by low P concentrations. *A. platensis* appeared to be much more effective in releasing P from zeolite when compared to *C. vulgaris*. It is known that in natural systems the sediments are sinks of various nutrients either in the organic or inorganic (Fe/Al and Ca precipitations) form. Microalgae and in general benthic microorganisms induce the nutrient release from the sediments [18,19]. However, it is not known if the P fractions in the sediment are bound to the clastic materials of the sediments. As far as the authors know, there are no data about the biological desorption of nutrients from zeolite or other natural clastic materials by microalgae. Considering that both species investigated, could uptake more P than could be spontaneously desorbed physically/chemically in the cultivation media, it is assumed that the microorganisms could desorb the P producing (excreting) some biomolecules that are capable to release the bounded P. The main mechanism of P sorption onto Ca(OH)_2_ pre-treated zeolite is the formation of Ca–P precipitation (data in preparation for submission) on the zeolite’s surface. So the biological desorption of P should be due to the production of various biomolecules, which disconcert the Ca–P complex. The excretion of organic acids or organic ligands in higher plants in the rhizosphere for the enhancement the bioavailability of P is well known [20], however some analogous for microalgae and cyanobacteria has not been described so far. An additional possible way of P desorption could be the P concentration gradient over time [21], *i.e.*, the disturbing of the sorption equilibrium between the solid and aqueous phase due to the cell uptake of P. Although P was absolutely taken-up by the microorganisms even from the first days, different P desorption percentages observed, a fact that suggests that this way of P desorption did not contributed significantly to the P uptake.

When the applied P-loaded zeolite corresponded to limited P source, then the two species had a different behavior regarding the biomass production, the biomass composition and the intracellular P content (Figure 4 and Figure 6). Although both species have been studied extensively in synthetic media and wastewater under various nutrient concentrations [22,23,24,25,26,27], there are no adequate data to compare the behavior of microalgal and cyanobacteria concerning the relationship between growth and intracellular nutrient concentration.

Based on the results of the present study, under P limited conditions, *A. platensis* can recover and use P more efficiently for biomass production than *C. vulgaris*. This capability of *A. platensis* to grow at the expense of the intracellular P was demonstrated in previous study [14]. That P biomass content increases by the increase of applied P in cultures of *C. vulgaris* was previously shown by Fitzgerald and Nelson [28]. This indicates that *A. platensis* prefer to synthesize biomass at the expense of intracellular P, while *C. vulgaris* prefers to increase the intracellular P. However, this point deserves more research.

Nutrient starvation effect on the biochemical composition of microalgae and cyanobacteria, including *C. vulgaris* and *A. platensis*, has been extensively studied (see for instance the reviews Hu, *et al.* [29] and Markou, *et al.* [30]). Phosphorus is an essential nutrient participating in vital organic molecules such as RNA, DNA, ATP and phospholipids, [31] and the effect of P starvation on carbohydrates or lipid content of *C. vulgaris* contradictory results are reported in the literature, indicating that there are some other parameters which co-effect the alteration of the biomass composition inducing the accumulation of either carbohydrates or lipids [30,32,33]. In contrast, P starvation in *A. platensis* is demonstrated that induces the accumulation mainly of carbohydrates [30].

Zeolite is well known that adsorbs heavy metals through ion-exchange [34] and additionally, due to its porous structure could be act as an attachment medium for various microorganisms contained in wastewater [35]. Regarding the produced biomass quality using the technique of zeolite nutrient separation, this point requires further investigation to ensure that zeolite would not transfer and release heavy metals and/or pathogens or any other contamination compound from the wastewater to the cultivation medium.

## 4. Experimental Section

### 4.1. Microorganisms

The green microalga *C. vulgaris* SAG 211-11b and the cyanobacterium *A. platensis* SAG 21.99 used in the study were obtained from SAG (Sammlung von Algenkulturen der Universität Göttingen, Lower Saxony, Germany). The green alga *C. vulgaris* was cultivated in modified BG-11 medium (initial pH 7) with the following composition: 20 mg/L Na_2_CO_3_, 2.5 g/L NaNO_3_, 0.2 g/L K_2_SO_4_, 36 mg/L CaCl_2_, 75 mg/L MgSO_4_·7H_2_O, 6 mg/L citric acid, 6 mg/L ammonium ferric citrate green, 1 mg/L Na_2_EDTA and 1.0 mL of trace elements: 2.86 g/L H_3_BO_3_, 20 mg/L (NH_4_)_6_Mo_7_O_24_, 1.8 g/L MnCl_2_·4H_2_O, 80 mg/L CuSO_4_·5H_2_O and 220 mg/L ZnSO_4_·7H_2_O.

*A. platensis* was cultivated in modified Zarrouk medium which had the following composition: 16.8 g/L NaHCO_3_, 2.5 g/L NaNO_3_, 1.0 g/L K_2_SO_4_, 1.0 g/L NaCl, 40 mg/L CaCl_2_, 80 mg/L Na_2_EDTA, 200 mg/L MgSO_4_·7H_2_O, 10 mg/L FeSO_4_·7H_2_O and 1.0 mL of trace elements as above. In both culture media, the phosphate (K_2_HPO_4_) was omitted, and as P source P-loaded zeolite was used (see 4.2 experimental set-up).

### 4.2. Experimental Set-Up

#### 4.2.1. Enrichment of Zeolite with P

The natural zeolite used was clinoptilolite with purity of 92%–94%, particle size of 4–6 mm and with the following chemical composition: SiO_2_ 64.88%, Al_2_O_3_ 12.99%, CaO 3.26%, Fe_2_O_3_ 2.00%, MgO 1.07%, Na_2_O 0.95%, K_2_O 0.89%, TiO_2_ 0.37%. The zeolite prior to the experiments was washed several times with DI water to washout unwanted particles and to increase its purity. Zeolite after washing was dried overnight at moderate temperature (70 °C).

Natural zeolite displays a very low P sorption capability, because its surface has negative electrical charge, which repulses the uptake of anions such as orthophosphate [9]. Therefore for the enhancement of P sorption capability, zeolite was chemically pre-treated using Ca(OH)_2_ as the modifying agent. For the modification of zeolite 100 g/L of natural zeolite was added in 1 M of Ca(OH)_2_ solution and agitated (100 rpm) for 24 h at room temperature.

After the modification of zeolite, its enrichment was conducted in sealed 500 mL conical flasks, which were placed on an agitation plate and were agitated for 72 h at room temperature. The enrichment of the pre-treated zeolite with P was performed by the addition of 20 g/L zeolite in artificial wastewater composed by (modified from He, *et al.* [36]): NH_4_Cl, 2000 mg-N/L; K_2_HPO_4_, 200 mg-P/L; NaNO_3_, 100 mg-N/L; CaCl_2_·2H_2_O, 150 mg/L; KCl, 300 mg/L; NaCl, 300 mg/L; MgSO_4_·7H_2_O, 1600 mg/L and 1 mL/L trace metals (same as the BG-11 and Zarrouk media).

The sorption (enrichment) onto zeolite, *i.e.*, the amount of the sorbate sorbed onto zeolite at equilibrium conditions, *q_e_* (mg/g), was calculated with the following equation:

(1) $$q_{e}=\frac{C_{o}-C_{e}}{C_{s}}$$

where *C_o_* (mg/L), *C_e_* (mg/L), and *C_s_* (g/L) are the initial P concentration, the P concentration at equilibrium conditions, and the zeolite concentration in the solution, respectively. The zeolite was finally enriched with 10.3 mg-P/g. In addition to the P, ammonium was also sorbed at 8.5 mg-N/g. However, the present work focused only in the utilization of P as nutrient for the production of microalgal and cyanobacterial biomass. The zeolite prior to the experiments was rinsed several times with DI water.

#### 4.2.2. P Desorption Kinetics

In a series of experiments the P desorption of the P-enriched zeolite were investigated. 0.1–2.5 g/L of P-enriched zeolite was placed in 0.06 M-1 HCl, 1 M NaOH, 1 M KCl and in modified BG-11 and Zarrouk medium (without K_2_HPO_4_). Samples of 40 mL were placed in 50 mL plastic centrifuge tubes and agitated by an agitation plate. Desorption of P from zeolite to the solution was calculated as follow:

(2) $$\text{Desorption}\;\left(\%\right)=\frac{\text{P}\;\text{in}\;\text{solution}}{\text{Zeolite}\;\text{P}\;\text{load}}\times 100$$

#### 4.2.3. P-Recovery Calculations

The amount of P (mg-P) that was taken up by *C. vulgaris* and *A. platensis* was assumed to be equal to the intracellular P content. P-recovery was calculated according to the following equation:

(3) $$\text{P}–\text{recovery}\;(\text{\%})=\frac{\text{Intracellular}\;\text{P}}{\text{Zeolite}\;\text{P}\;\text{load}}\times 100$$

Explicitly, P-recovery was the ratio of P taken-up by the microorganisms and the amount of P added to the cultures as P-loaded zeolite.

#### 4.2.4. Cultivation of *C. vulgaris* and *A. platensis*

After the P enrichment of the zeolite, the P-loaded zeolite was washed several times with DI water in order to ensure that the P as P source for the cultivation of the microorganisms derived from the zeolite and not from wastewaters residuals. Zeolite was added to already inoculated cultures with *C. vulgaris* or *A. platensis*. Due to gravity zeolite settled to the bottom of the photobioreactor (PBR). The aeration of the culture was able to suspend biomass but not able to suspend zeolite granules in the culture media and did not generate any turbidity in the medium, so the mass of zeolite added to the cultures did not affect the photosynthetic efficiency of the cultures. During the cultivation no significant attachment of the biomass onto zeolite was observed.

*C. vulgaris* and *A. platensis* were cultivated in batch mode in the modified media mentioned before. The modification of the media refers to the replacement of K_2_HPO_4_ with P-loaded zeolite as the P source. The initial pH of the media was 7 and 8.5 for *C. vulgaris* and *A. platensis*, respectively. However, due to photosynthesis and the release of OH^−^, the pH in both media reached 9.5. In the first experimental series five concentrations of P-loaded zeolite were used, namely 0.15, 0.20, 0.25, 0.30, 0.50 and 1.00 g/L. This concentration range was chosen in order to provide limited mass of P in order to investigate the capability of each microorganism to utilize the onto-zeolite sorbed P. Considering the high requirements of *C. vulgaris* and *A. platensis* to nitrogen, the effect of ammonium, which was sorbed in the zeolite was considered as negligible. In a second experimental series, P-loaded zeolite in concentration of 5 g/L was used to investigate biomass production with replete P source.

The experiments were carried out in 400 mL PBR. The working volume was set on 250 mL. The cultures were aerated, in order to be agitated, with filtered air provided by a membrane air pump. The cultivation was carried out in air-conditioned room and culture temperature was kept constant at 30 °C (±2 °C). Light intensity (measured in the middle of the PBR) was set at 6 klux for the first experimental series, while for the second one the light intensity was set at 9.5 klux. Light was provided through a 57 W cool fluorescence tube-lamp on the one side of the PBR at a photoperiod day:night of 16 h:8 h. Cultures were carried out in duplicates.

### 4.3. Analytical Methods

Dry algal biomass was measured indirectly by spectrophotometry at 560 and 750 nm for *A. platensis* and *C. vulgaris*, respectively. Proteins were determined according to the Lowry method using bovine albumin as standard [37], total lipids according to the sulfo-phospho-vanillin reaction method, using corn-oil as standard [38], carbohydrates by the phenol-sulfuric acid method using d-glucose as standard [39] and pigments were extracted with hot 90% methanol and their concentrations were calculated using the equations given by Lichtenthaler [40]. All biomass composition analyses were performed after the washing of the samples for several times with DI water. Ammonium was measured with the phenate method according to Solorzano [41]. Phosphate was determined with the ascorbic acid method [42] and intracellular P with the same method after digestion of biomass with potassium persulfate at 70 °C for 24 h. All spectrophotometric determinations were carried out on a Cadas 30 (Dr Bruno Lange GmbH, Berlin, Germany) spectrophotometer and analyses were carried out at least in triplicates.

## 5. Conclusions

The separation and recovery of P from wastewater by means of zeolite sorption and its use as phosphorus (P) source for the cultivation of microalgae and cyanobacteria is a promising approach for the production of biomass avoiding the direct use of wastewaters as cultivation medium. In this study chemically pre-treated zeolite was used as the sorbent for the separation of P from synthetic wastewater. Then the P-loaded zeolite was applied as P source for the cultivation of *Chlorella vulgaris* and *Arthrospira* (Spirulina) *platensis*. At the experimental series in which P was limited (applying P-loaded zeolite with concentrations of 0.15–1 g/L), the two species investigated displayed quite different behavior regarding their growth and biomass composition. Both species displayed an interesting capability of desorbing biologically the zeolite’s bounded P. At higher degree of P limitation, both species desorbed P with higher efficiency. Between the two species, *A. platensis* was more efficient to desorb and use P from zeolite. Both species displayed a good growth when P-loaded zeolite concentration increased to 5 g/L. At this concentration P was adequate to support growth.
